# Determinant Causes of Limb Amputation in Ethiopia: A Systematic Review and Meta-Analysis

**DOI:** 10.4314/ejhs.v33i5.19

**Published:** 2023-09

**Authors:** Bickes Wube Sume, Soressa Abebe Geneti

**Affiliations:** 1 Department of Anatomy, School of Medicine, College of Health Sciences, Addis Ababa University, Addis Ababa, Ethiopia

**Keywords:** Amputation, Determinant causes, Ethiopia

## Abstract

**Background:**

Most amputees suffered from lack of rehabilitation services and went on streets as glorified beggars. However, there is a paucity of information about determinant causes of amputation in Ethiopia. Therefore, this systematic review and meta-analysis was conducted to estimate pooled prevalence of limb amputation and its determinant causes in Ethiopian population.

**Methods:**

Worldwide databases such as PubMed/MedLine, Web of Science, CINAHL, Embase, Scopus, and Science Direct were searched to retrieve pertinent articles. Grey literatures were also looked in local and national repositories. Microsoft excel was used to extract data which were exported to stata version 14.0 for analysis. Cochrane Q and I^2^ tests were used to assess heterogeneity. Egger's and Begg's tests were employed to assess reporting biases. Random effect meta-analysis model was applied to estimate pooled prevalence.

**Results:**

Twenty-one qualified studies with 18,900 study participants were reviewed. Pooled prevalence of limb amputation was 31.69%. Lower extremity amputation (LEA) accounted for 14.41%, and upper extremity amputation (UEA) took 10.53% (6.50, 14.53). Above knee amputations (2.50 %) were common orthopedic operations whereas ray amputations (0.03%) were the least orthopedic procedures of LEA. Above elbow amputations (2.46%) were common from UEA while shoulder disarticulations (0.02%) were the least orthopedic surgical procedures. The major causes of limb amputations were trauma (11.05%), diabetic foot ulcer (9.93 %), traditional bone setters (24.10%) and burn (10.63%).

**Conclusions:**

Lower extremity amputations were common orthopedic surgical procedures. Major determinant causes were trauma, diabetic foot ulcer, traditional bone setters and burn.

## Introduction

Amputation is a surgical operation for removing a limb when limb recovery is impossible or when the limb is dead or nonfunctional, endangering the patient's life (1). It is a frequent and mutilating orthopedic surgical procedure which alters the bodily image and causes substantial functional deficits (2). Amputation is both a life-saving and life-altering surgical intervention. Despite the fact that amputation is done to save life, patients confront difficulties such as limitations in physical functionality, prosthesis use, intimate personal connection, employment status and lifestyle (3).

Limb amputation (LA) is continuing to be a critical public health issue and causing significant financial depletions. Amputation causes differ between and within countries (1, 3, 4). According to surveys of specialized child amputee clinics in affluent nations, roughly 60% of youth amputations are due to congenital limb defects and 40% are due to acquired disorders (5). Tumors and traumas are frequently described as the primary causes in sub-Saharan Africa, but peripheral vascular disorders are the most common explanations in industrialized countries (6, 7).

In terms of amputation site, different studies revealed different results, but lower extremity amputations (LEA) accounted for 79% of all limb loss discharges from hospitals (8). Identifying the appropriate level of amputation is critical in order to decide whether amputation is an appropriate form of treatment or not (9). It is not the epilogue of a story, instead, it's the beginning of a difficult life. Levels of LA are largely decided by pathological, anatomical, surgical, prosthetic and patient preferences (10). Each of these considerations would play a role in the decision to ablate the limb at a specific level with a varying weight depending on the clinical situation (10). In descending order, the patterns of LA in low income countries were trans-tibial, trans-femoral, trans-radial and trans-humeral (11). The majority of amputee patients in developed countries were over 60 years old, and vascular issues accounted for 80-90% of LEA (12).

Yet, in developing countries, the majority of amputees were young and the primary causes of LA were different from hospital to hospital (13). Sadly, patients in developing countries frequently appeared late when limb salvage was no longer a possibility (14). The loss of a limb by any individual, particularly in developing nations where prosthetic services are inadequate, can have far reaching economic, social and psychological consequences for the patients, their families and the society as a whole (15, 16).

In developing countries, most LA are preventable by tightening traffic restrictions, public health education, proper care of common illnesses, strong healthcare regulation and provision of affordable healthcare services (2, 17). The progress in components, fitting procedures, suspension systems and digital controls have led to noteworthy enhancements in the progress of prosthetic limbs for people who have suffered from amputations of upper or lower limbs (18). However, the lack of data to back current methods for addressing extremity traumas has led to the neglect of emergency and trauma care quality in low and middle income countries (19).

In Ethiopia, for instance, amputation is the sole viable treatment for late effects of trauma, diabetes, tumors and other persistent infections (20). Ethiopians suffered from lack of effective rehabilitation services for these wretched patients who end up on the streets as glorified beggars. However, there is no summative evidence of the leading causes of these disabled conditions. Therefore, this systematic review and meta-analysis was conducted to estimate the pooled prevalence of limb amputation and its determinant causes in the Ethiopian population.

## Materials and Methods

**Study design and setting**: This systematic review and meta-analysis was conducted to determine the magnitude and determinant causes of limb amputation in Ethiopia.

**Searching strategies**: Based on the study's eligibility criteria, we reviewed a variety of literatures including both published and unpublished studies. To ensure scientific rigor, the Preferred Reporting Items for Systematic Reviews and Meta-Analysis Procedure (PRISMA-P) guideline was adopted (21). We searched PubMed/MedLine, Web of Science, CINAHL, Embase, Scopus, Cochrane Library, Google Scholar and Science Direct to retrieve pertinent articles. Grey literatures were also looked in local and national repositories. The protocol was registered in Prospero (CRD42023388895). The key terms considered in developing the search strategy for the databases were prevalence, magnitude, amputations, limb loss, upper limb, lower limb, extremities, determinant, causes, trauma, gangrene, diabetic foot ulcer, road traffic accidents, peripheral vascular disorders, tumors, malignancies, burn, machine injuries and work-related accidents in the context of Ethiopia. To search electronic databases, the key terms were joined using Boolean operators.

All studies related to limb amputations conducted in Ethiopia regardless of publication date and study design, evidences which reported limb amputation and its determinant causes were included. The initial appraisal started by reading the titles and abstracts based on the eligibility criteria. The entire articles were read when the studies were found relevant for our review, we read the entire texts. Any papers that were not completely accessible at the time of our search were excluded after at least two attempts to contact the principal investigator through email. Furthermore, after analyzing their full texts, studies that did not report our outcome of interest were also excluded.

**Quality assessment**: Endnote (version X7) software was used to manually purge duplicate articles from database search results. The quality of each studies were evaluated using the Newcastle-Ottawa quality assessment tool scale (22). Each study was reviewed critically by two independent reviewers (BWS and SAG). The review's disputes were resolved through discussion.

**Data extraction**: Standardized data extraction spreadsheet was used to extract data. The data extraction spreadsheet was tested on 5 randomly selected publications and then adjusted as needed. The data extraction form comprised study parameters such as (1) author's name, publication year, study area, region, study design and sample size; (2) prevalence of limb amputations: upper limb and lower limb; (3) determinant causes of limb amputations: trauma, tumor, diabetic foot ulcer, peripheral vascular diseases, traditional bone setters, burn and others.

**Outcome of the study**: The first outcome of the study was to estimate pooled prevalence of LA in the Ethiopian population. The second outcome of the study was to identify the determinant causes of LA.

**Data analysis**: The extracted data were exported to stata software version 14 for analysis. To declare the presence of publication biases, both Egger's and Begg's tests were used with a p-value less than 0.05 as a cut-off point (23). Heterogeneity across studies was assessed using the Cochran Q statistic with the inverse variance (I^2^) of 30 to 60%, 50 to 90%, and 75 to 100% with moderate, substantial, and significant heterogeneity across individual studies (24). The funnel plot was used to demonstrate the presence of heterogeneity. Subgroup analysis and meta-regression were used to investigate potential differences between the studies. Using the random effects meta-analysis (DerSimonian and Laird) model, the findings were presented as a forest plot with an odds ratio and 95% confidence intervals.

## Results

**Figure 1:**
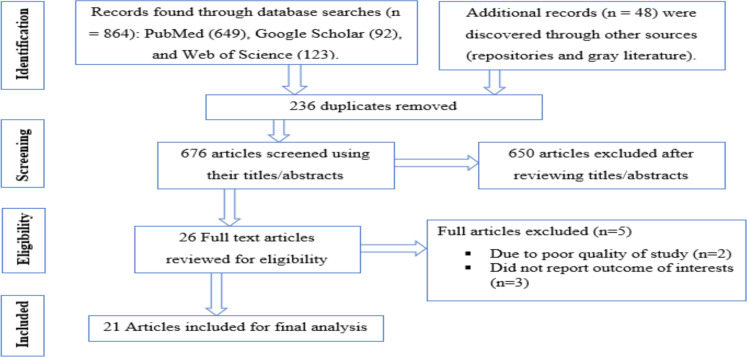
PRISM flow chart for limb amputation in Ethiopia, 2023

**Study selection and search results**: From 20 December 2022 to 30 February 2023, a total of 912 studies were identified through electronic searches (864 articles) and manual searches (48 articles). Two hundred and thirty-six studies were excluded due to duplicated queries. Following a review of their titles and abstracts, 650 studies were removed as irrelevant. The remaining 26 full-text articles were evaluated for eligibility. Finally, 21 studies met the eligibility criteria and were included in the current systematic review and meta-analysis (Fig. 1). Five articles, however, did not meet the inclusion criteria and were excluded with clear reasons (25-29).

**Characteristics of selected articles**: A total of 21 studies with 18,900 participants were included to estimate the pooled prevalence of LA and its determinant causes in the Ethiopian population. Of these studies, 11 studies were conducted in Addis Ababa, 3 studies in Southern Nations, Nationalities and Peoples (SNNP), 2 studies in Oromia, 2 studies in Tigray; and a single study was reported from Amhara, Harar and Dire Dawa. Thirteen studies were conducted in all age groups, 6 studies in adults and 2 studies in pediatric age group (Table 1).

**Table 1:** Characteristics of selected studies of limb amputation in Ethiopia, 2023

Authors' name	Publication year	Study region	Sample size	AG	LA	LEA	UEA	Male	Female	Quality score
Bekele F, Chelkeba L (30)	2020	Oromia	115	Ad	35	35	0	19	16	9
Yasin SM et al (31)	2018	Addis Ababa	99	ped	102	50	52	75	24	8
Gizaw M et al (32)	2015	Addis Ababa	130	aag	23	23	0	NR	NR	9
Amogne W et al (33)	2011	Addis Ababa	196	Ad	92	92	0	NR	NR	9
Lester FT (34)	1995	Addis Ababa	2250	aag	43	43	0	NR	NR	8
Tibebu NS et al (35)	2021	Amhara	405	Ad	129	NR	NR	NR	NR	9
Takele G (gr)	2018	SNNP	384	aag	61	26	35	39	22	8
Inki NG et al(36)	2018	Oromia	850	aag	39	39	0	27	12	8
Dessie MM (37)	2009	Dire Dawa	1248	aag	68	12	56	NR	NR	9
Elias A, Tezera C(38)	2005	Addis Ababa	7151	aag	197	31	162	NR	NR	8
Mulatu D et al(39)	2022	Addis Ababa	241	aag	19	NR	NR	NR	NR	8
Gebreslassie B et al (40)	2018	Tigray	87	aag	87	51	36	68	19	9
Amin A et al (gr)	2020	Harar	3433	aag	110	85	25	81	29	8
Srahbzu M et al (41)	2018	Addis Ababa	407	Ad	38	25	13	NR	NR	8
Dessie M(42)	2004	Addis Ababa	110	aag	110	83	27	83	27	8
Ahmed E(43)	2010	Addis Ababa	253	Ad	151	0	151	NR	NR	9
Eshete M (44)	2005	SNNP	49	aag	49	NR	NR	NR	NR	9
Getu J, Bayisa ET(45)	2017	SNNP	280	aag	28	NR	NR	NR	NR	8
Seyoum N et al (gr)	2021	Addis Ababa	102	Ad	68	NR	NR	26	42	8
Ahmed E, Chaka T(46)	2006	Addis Ababa	602	aag	126	0	126	NR	NR	8
Hagos M (47)	2017	Tigray	508	Ped	8	NR	NR	NR	NR	8

AG = Age group, LA = Limb amputation, LEA = Lower extremity amputation, UEA = Upper extremity amputation, NR = Not reported, Ad = Adult, Ped = Pediatric, aag = All age group, gr =grey literature

**Figure 2:**
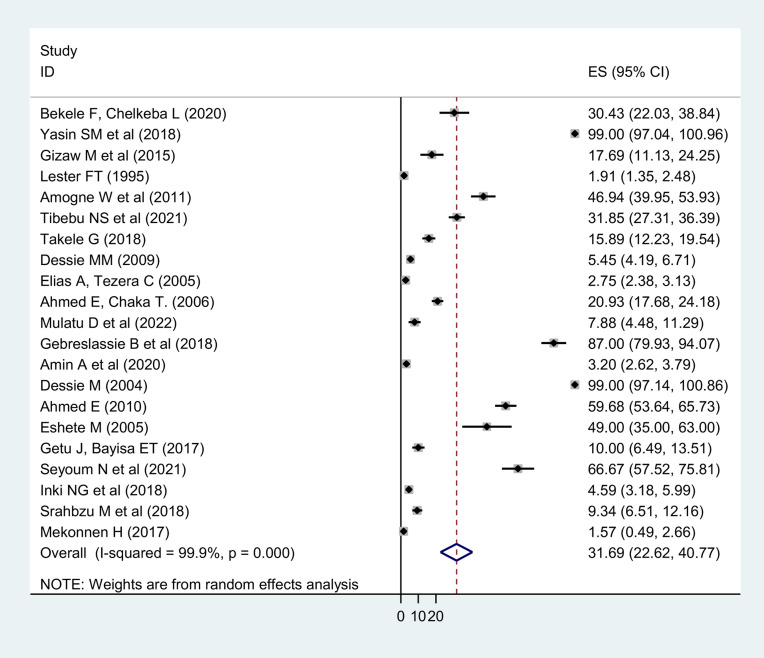
Pooled estimate of limb amputation in Ethiopia, 2023

**Pooled prevalence of limb amputation**: The pooled prevalence of LA was 31.69% (95 % CI: 22.62, 40.77) (Fig. 2). LEA accounted for 14.41% (95 % CI: 8.39, 20.43). UEAs were also estimated to account for 10.53% (6.50, 14.53) of LA. Above knee amputations (AKA) (2.50 % (95 % CI: 0.76, 4.23)) were the most common surgical procedure among LEAs. Ray amputations (RA) (0.03% (95 % CI: -0.22, 0.28)), on the other hand, were the least common orthopedic procedures from LEAs. In terms of UEAs, above elbow amputations (AEA) (2.46% (95 % CI: 0.64, 4.28) were the most common while shoulder disarticulation (SDA) (0.02% (95 % CI: -0.23, 0.27)) and elbow disarticulation (EDA) (0.03% (95 % CI: -0.22, 0.28)) were the least common orthopedic surgical procedures.

**Figure 4:**
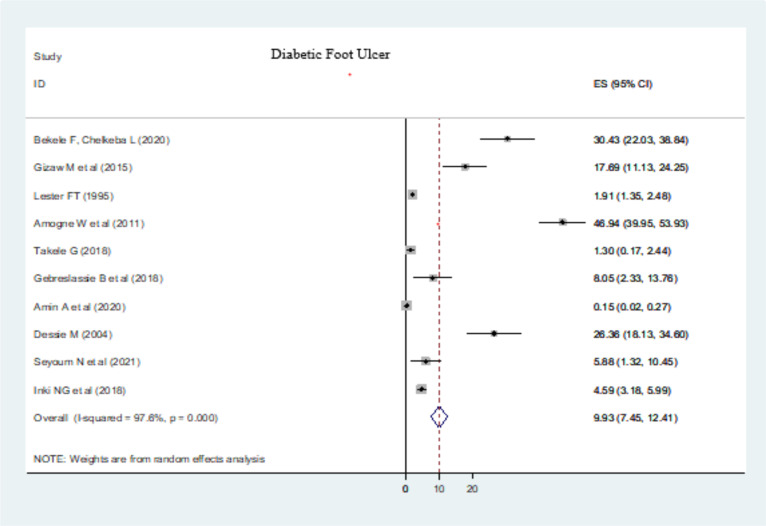
Diabetic foot ulcer as a determinant causes of limb amputations in Ethiopia, 2023

**Figure 5:**
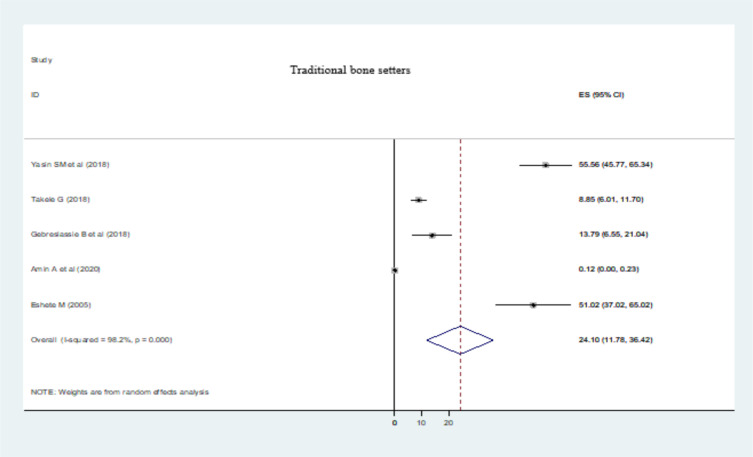
Traditional bone setters as a determinant causes of limb amputations in Ethiopia, 2023

**Determinant causes of limb amputation**: The major determinant causes of LA were trauma (11.05% (95% CI: 8.95, 13.15)) (Fig. 3), diabetic foot ulcer (DFU) (9.93 % (95% CI: 7.45, 12.41)) (Fig. 4), and traditional bone setters (TBS) (24.10 % (95% CI: 11.78, 36.42)) (Fig. 5). Other indications of extremity amputations were burn (10.63 % (95% CI: 0.92, 20.33)), tumor (6.56% (95% CI: 3.66, 9.45)), peripheral vascular disease (PVD) other than diabetics (4.13% (95% CI: 1.92, 6.34)), road traffic accidents (RTA) (0.61% (95% CI: -0.02, 1.25)), machine injuries (3.17% (95% CI: 0.24, 6.1)), and others (19.12% (95% CI: 11.83, 26.40)). From DFA causes, type I diabetes mellitus (DM) constituted 0.03% (95% CI: -0.10, 0.10) of LA whereas type II DM took 2.21% (95% CI: 0.72, 3.69) of extremity amputations as a determinate condition.

**Subgroup analysis:** Based on the subgroup analysis, the highest prevalence of LA was reported from Tigray (44.21 % (95% CI: -39.50, 127.93)) followed by Addis Ababa (39.20% (95% CI: 21.64, 56.76)) and Amhara region (31.85% (95% CI: 27.31, 36.39)). The SNNP and Oromia regions had 21.80% (95% CI: 10.44, 33.15) and 17.17% (95% CI: -8.15, 42.49) of limb amputations respectively. However, relatively the smallest number of extremity amputated cases were reported from the Harar (3.20 % (95% CI: 2.62, 3.79)) and Dire Dawa (5.45% (95% CI: 4.19, 6.71)) city administrations. Moreover, the highest prevalence of LA was revealed in pediatrics age group (50.28% (95% CI: -45.19, 145.76)) followed by adult age group (46.99% (95%CI: 33.05, 60.93)).

**Publication bias and meta-regression**: We used a funnel plot to assess publication bias, and the scatter plots of each study became less condensed near zero. As a result, both Begg's and Egger's tests were considered as confirmatory tests. Begg's and Egger's tests yielded p-values of 0.53 and 0.47 respectively, indicating that there were no publication biases. A meta-regression analysis also revealed that age groups (p = 0.27), publication years (p = 0.43), and study regions (p = 0.28) did not explain heterogeneity.

**Subgroup analysis**: Based on the subgroup analysis, the highest prevalence of LA was reported from Tigray (44.21 % (95% CI: -39.50, 127.93)) followed by Addis Ababa (39.20% (95% CI: 21.64, 56.76)) and Amhara region (31.85% (95% CI: 27.31, 36.39)). The SNNP and Oromia regions had 21.80% (95% CI: 10.44, 33.15) and 17.17% (95% CI: -8.15, 42.49) of limb amputations respectively. However, relatively the smallest number of extremity amputated cases were reported from the Harar (3.20 % (95% CI: 2.62, 3.79)) and Dire Dawa (5.45% (95% CI: 4.19, 6.71)) city administrations. Moreover, the highest prevalence of LA was revealed in pediatrics age group (50.28% (95% CI: -45.19, 145.76)) followed by adult age group (46.99% (95%CI: 33.05, 60.93)).

**Publication bias and meta-regression**: We used a funnel plot to assess publication bias, and the scatter plots of each study became less condensed near zero. As a result, both Begg's and Egger's tests were considered as confirmatory tests. Begg's and Egger's tests yielded p-values of 0.53 and 0.47 respectively, indicating that there were no publication biases. A meta-regression analysis also revealed that age groups (p = 0.27), publication years (p = 0.43), and study regions (p = 0.28) did not explain heterogeneity.

## Discussion

Amputation is the oldest surgical procedure by orthopedists and other surgeons to save the lives of many patients (48). It should be the first step in the rehabilitation of a patient with a nonfunctional limb rather than being the final step as a treatment (49). The contributing factors and motifs of LA differ from hospital to hospital within a country. Thus, the aim of this systematic review and meta-analysis was to estimate the pooled prevalence of LA and its determinant causes in the population of Ethiopia. The pooled prevalence of LA was found 31.69% (95% CI: 22.62, 40.77) with 14.41% LEA and 10.53% UEA. Our findings were in line with the findings of studies conducted in Germany, 33.8% (50), and Norway, UEA 11.6% (51).

On contrary, our findings were lower than the findings of studies done in Nigeria (LEA:74.2%, UEA: 25.8%) (52), Nepal (LEA: 73.58%), Cameroon (LEA:70.3%, and UEA: 29.7%) (1, 53). In Nepal, the major causes of LA were RTA (74.29%), burn (29.54%), and congenital limb deformities (22.72%). RTA (0.61%) and burn (10.63 %) were also decisive factors of LA in our study. This might be due to inadequately treated fractures as well as vehicle accidents and other motorized machineries (18).

The relative pervasiveness of lower limb amputations in the current study was consistent with previous reported studies in Tanzania (14) and Turkey (54). The possible explanations might be due to the fact that lower extremity surgical complications are more common in individuals with lower incomes because they are more likely to live in rural areas with few or no healthcare care options (55). Moreover, above knee amputation (AKA) and below knee amputation (BKA) were found the most common levels of amputation. This was similar with the findings of studies conducted in Tanzania and Nigeria (14, 56).

Trauma was the major determinant causes of amputation in the present study. This agreed with studies conducted in Nigeria (57), Turkey (54), and Kenya (6). However, other previous studies by Ofiaeli RO (58), Dada AA, Awoyomi BO (56), and Omoke N et al (57), DFU was reported as a major detriment factor for limb loss. Our findings also varied from high-income countries where PVD were the leading cause of extremity amputations (7).

In the current study, DFU and other PVD were causes of extremity amputations. DM and PVD amputations impose a significant financial strain on individuals, family members and communities at large. More than 55% of those who have amputations as a result of diabetes or PVD were permanently disabled (59). Those who have AKA, in particular, never regain ambulatory status (60).

When function is the paramount concern, amputation should be executed at the bottom of the priority list (61). This indicates the lower the level of amputation the better the amputee's performance. On the other hand, it reduced rehabilitation needs, increased use and satisfaction with a prosthesis, provided a biomechanical advantage and reduced financial burden (62).

In our study, LA caused by improperly treated extremity injuries by TBS were encountered as a decisive factor for limb loss. TBS (“Wogesha”) is widely practiced throughout Ethiopia, but its perks and atrocities have never been reported (31). About 85% of fracture patients in Africa are seen by TBS before going to hospitals (63). However, their mode of health services ought not to be missed. Economic inequality, lack of schooling, ethos of the community, indigenous practices and vitriolic banquet in health care settings attributed to the resumption of their abhorrent practice (64, 65). Similar role of TBS in LA were also reported in other developing countries (15, 16, 56). This might not strange that trauma is the most common cause of LA in Ethiopia.

The current study also revealed that the burden of amputation and limb stump affect people of all ages. No age group was exempted from this surgical procedure. The magnitude of amputees was relatively high in pediatrics age group compared to adults. This might be due to the fact that children are more exposed to trauma and miserable conditions. This is supported by reported studies which revealed that trauma was the leading cause of amputation below 15 years of age (66, 67). This was also similar to other reported studies from Nigeria (58) and Kenya (6).

The subgroup analysis also revealed that the magnitude of LA varies greatly across the country. Tigray had the highest rate of LA followed by Addis Ababa, Amhara, SNNP, Oromia, Harar, and Dire Dawa in a descending order. The regional discrepancies might be attributed to early trauma management, occupational safety, restricted traffic rules and prevention of chronic disease complications like diabetics.

In conclusion, about one third of the cases had LA. LEA were most common orthopedic surgical procedures. AKA were major limb stumps from LEA whereas AEA were also most common surgical operations from UEA. The major determinant causes of LA were trauma, DFU, TBS and burn. The magnitude of LA was found high in pediatric age group compared to adults. The Tigray region had the highest number of limb amputees to other regions. The findings of this review alert future health care planning for the amputees including prosthesis, multidisciplinary rehabilitation services, occupational rehabilitation and preventive measures.

## References

[1] Paudel B, Shrestha B, Banskota A (2005). Two faces of major lower limb amputations. Kathmandu University medical journal.

[2] Nwosu C, Babalola MO, Ibrahim MH, Suleiman SI (2017). Major limb amputations in a tertiary hospital in North Western Nigeria. African health sciences.

[3] Østlie K, Lesjø IM, Franklin RJ, Garfelt B, Skjeldal OH, Magnus P (2012). Prosthesis use in adult acquired major upper-limb amputees: patterns of wear, prosthetic skills and the actual use of prostheses in activities of daily life. Disability and Rehabilitation: Assistive Technology.

[4] Banza L, Mkandawire N, Harrison W (2009). Amputation surgery in children: an analysis of frequency and cause of early wound problems. Tropical doctor.

[5] Aitken GT (1963). Surgical amputation in children. JBJS.

[6] Ogeng'o JA, Obimbo MM, King'ori J (2009). Pattern of limb amputation in a Kenyan rural hospital. International orthopaedics.

[7] Hussain MA, Al-Omran M, Salata K (2019). Population-based secular trends in lower extremity amputation for diabetes and peripheral artery disease. CMAJ.

[8] Dillingham TR, Pezzin LE, MacKenzie EJ (2002). Limb amputation and limb deficiency: epidemiology and recent trends in the United States. Southern medical journal.

[9] MacKenzie EJ, Bosse MJ, Castillo RC (2004). Functional outcomes following trauma-related lower-extremity amputation. JBJS.

[10] Murdoch G (1967). Levels of amputation and limiting factors. Annals of the Royal College of Surgeons of England.

[11] Soomro N, Bibi R, Ahmed SI, Kamran B, Minhas MA, Siddiqui KY (2013). Epidemiology of amputation; Low resource community: Sindh Province, Pakistan (october 2007-june2012). The Professional Medical Journal.

[12] Rommers G, Vos L, Groothoff J, Schuiling C, Eisma W (1997). Epidemiology of lower limb amputees in the north of the Netherlands: aetiology, discharge destination and prosthetic use. Prosthetics and Orthotics International.

[13] Thanni L, Tade A (2007). Extremity amputation in Nigeria—a review of indications and mortality. The Surgeon.

[14] Chalya PL, Mabula JB, Dass RM (2012). Major limb amputations: A tertiary hospital experience in northwestern Tanzania. Journal of orthopaedic surgery and research.

[15] Yinusa W, Ugbeye M (2003). Problems of amputation surgery in a developing country. International orthopaedics.

[16] Nwankwo O, Katchy A (2004). Surgical limb amputation: A five-year experience at Hilltop Orthopedic Hospital Enugu, Nigeria. Nigerian Journal of Orthopaedics and Trauma.

[17] Bediako-Bowan AA, Adjei GO, Clegg-Lamptey JN, Naaeder SB (2017). The burden and characteristics of peripheral arterial disease in patients undergoing amputation in Korle Bu Teaching Hospital, Accra, Ghana. Ghana Medical Journal.

[18] Esquenazi A (2004). Amputation rehabilitation and prosthetic restoration. From surgery to community reintegration. Disability and rehabilitation.

[19] Kruk ME, Gage AD, Arsenault C (2018). High-quality health systems in the sustainable development goals era: time for a revolution. The Lancet global health.

[20] Spoden M, Nimptsch U, Mansky T (2019). Amputation rates of the lower limb by amputation level–observational study using German national hospital discharge data from 2005 to 2015. BMC health services research.

[21] Moher D, Liberati A, Tetzlaff J, Altman DG, PRISMA Group (2009). Preferred reporting items for systematic reviews and meta-analyses: the PRISMA statement. Annals of internal medicine.

[22] Modesti PA, Reboldi G, Cappuccio FP (2016). Panethnic differences in blood pressure in Europe: a systematic review and meta-analysis. PloS one.

[23] Begg CB, Mazumdar M (1994). Operating characteristics of a rank correlation test for publication bias. Biometrics.

[24] Chandler J, Hopewell S (2013). Cochrane methods-twenty years experience in developing systematic review methods. Systematic reviews.

[25] Bekele F, Berhanu D (2021). “Loss of a limb is not loss of a life”. Knowledge and attitude on diabetic foot ulcer care and associated factors among diabetic mellitus patients on chronic care follow-up of southwestern Ethiopian hospitals: A multicenter cross-sectional study. Annals of Medicine and Surgery.

[26] Biruk LW (2006). Permanent Civilian Musculoskeletal disability following injury-17 Year trends. East and Central African. Journal of Surgery.

[27] Mulugeta H, Zemedkun A, Getachew H (2020). Selective Spinal Anesthesia in a Patient with Low Ejection Fraction Who Underwent Emergent Below-Knee Amputation in a Resource-Constrained Setting. Local and Regional Anesthesia.

[28] Mohammed R, Van Griensven J, Ambaw AA (2022). Snake bite case management: a cohort study in Northwest Ethiopia, 2012-2020. J Infect Dev Ctries.

[29] Admassie D, Ayana B, Girma S (2015). Childhood limb fracture at Tikur Anbessa Specialised Hospital, Addis Ababa, Ethiopia. East Cent. Afr. J. surg.

[30] Bekele F, Chelkeba L (2020). Amputation rate of diabetic foot ulcer and associated factors in diabetes mellitus patients admitted to Nekemte referral hospital, western Ethiopia: prospective observational study. Journal of foot and ankle research.

[31] Yasin SM, Ayana B, Bezabih B, Wamisho BL (2018). Causes of pediatric limb amputations at Tikur Anbessa Specialized Hospital and the role of traditional bone setters (“Wogeshas”). Ethiop Med J.

[32] Gizaw M, Harries A, Ade S (2015). Diabetes mellitus in Addis Ababa, Ethiopia: admissions, complications and outcomes in a large referral hospital. Public Health Action.

[33] Amogne W, Reja A, Amare A (2011). Diabetic foot disease in Ethiopian patients: a hospital based study. Ethiopian Journal of Health Development.

[34] Lester F (1995). Amputations in patients attending a diabetic clinic in Addis Abeba, Ethiopia. Ethiopian medical journal.

[35] Tibebu NS, Desie T, Marew C, Wubneh M, Birhanu A, Tigabu A (2021). Health related quality of life and its associated factors among burn patients at governmental referral hospitals of amhara regional state, Northwest Ethiopia, 2020: Institutional based cross sectional study. Clinical, Cosmetic and Investigational Dermatology.

[36] Negasa Guta Inki GJ, Abdu Mohammed Abera (2018). Magnitude of lower limb amputation and associated factors among diabetic foot ulcer patients admitted to Adama hospital medical college from January 2013 to December 2017, Adama, Ethiopia. IJSAR.

[37] Dessie MM (2009). Major limb trauma in Eastern Ethiopia. East and Central African Journal of Surgery.

[38] Elias A, Tezera C (2005). Orthopedic and Major Limb Trauma at the Tikur Anbessa University Hospital, Addis Ababa, Ethiopia. East and central African journal of surgery.

[39] Mulatu D, Zewdie A, Zemede B, Terefe B, Liyew B (2022). Outcome of burn injury and associated factor among patient visited at Addis Ababa burn, emergency and trauma hospital: a two years hospital-based cross-sectional study. BMC emergency medicine.

[40] Gebreslassie B, Gebreselassie K, Esayas R (2018). Patterns and causes of amputation in Ayder Referral Hospital, Mekelle, Ethiopia: A three-year experience. Ethiopian journal of health sciences.

[41] Srahbzu M, Yigizaw N, Fanta T, Assefa D, Tirfeneh E (2018). Prevalence of depression and anxiety and associated factors among patients visiting orthopedic outpatient clinic at Tikur Anbessa specialized Hospital, Addis Ababa, Ethiopia, 2017. J Psychiatry.

[42] Dessie M (2004). Preventable amputations in Ethiopia. East and Central African Journal of Surgery.

[43] Ahmed E (2010). The management outcome of acute hand injury in Tikur Anbessa University Hospital, Addis Ababa, Ethiopia. East and Central African Journal of Surgery.

[44] Eshete M (2005). The prevention of traditional bone setter's gangrene. The Journal of bone and joint surgery British volume.

[45] Getu J, Bayisa ETH (2017). Factors Associated with Fracture and Its Outcome at Wolaita Sodo University Teaching and Referral Hospital, Wolaita Sodo, Southern Ethiopia. Journal of Biology, Agriculture and Healthcare.

[46] Ahmed E, Chaka T (2006). Prospective study of patients with hand injury: Tikur Anbessa University Teaching Hospital, Addis Ababa. Ethiopian medical journal.

[47] Hagos M (2017). Child injury admisions to a hospital in Ethiopia. Ethiopian medical journal.

[48] Sanders P, Wadey R, Day M, Winter S (2020). Narratives of recovery over the first year after major lower limb loss. Qualitative health research.

[49] Pinzur MS, Pinto M, Schon LC, Smith DG (2003). Controversies in amputation surgery. Instructional course lectures.

[50] Trautner C, Haastert B, Giani G, Berger M (1996). Incidence of lower limb amputations and diabetes. Diabetes care.

[51] Østlie K, Skjeldal OH, Garfelt B, Magnus P (2011). Adult acquired major upper limb amputation in Norway: prevalence, demographic features and amputation specific features. A population-based survey. Disability and rehabilitation.

[52] Ajibade A, Akinniyi O, Okoye C (2013). Indications and complications of major limb amputations in Kano, Nigeria. Ghana medical journal.

[53] Pisoh-Tangnyin C, Farikou I, Nonga BN (2010). Epidemiology of extremity amputations in yaounde-cameroon. Health Sciences and Disease.

[54] Dogan A, Sungur I, Bilgic S (2008). Amputations in eastern Turkey (Van): a multicenter epidemiological study. Acta orthopaedica et traumatologica turcica.

[55] Ashry HR, Lavery LA, Armstrong DG, Lavery DC, Van Houtum WH (1998). Cost of diabetes-related amputations in minorities. The Journal of foot and ankle surgery.

[56] Dada A, Awoyomi B (2010). Is the trend of amputation in Nigeria changing? A review of 51 consecutives cases seen at Federal medical centre Ebute Metta, Lagos, Nigeria. Nigerian Medical Journal.

[57] Omoke N, Nwigwe C (2016). Limb amputations in Abakaliki, South East Nigeria. African Journal of Medical and Health Sciences.

[58] Ofiaeli R (2001). Indications level and outcome of lower extremity amputations in Nnewi, Nigeria. Journal of Medical Investigation and Practice.

[59] Barnes JA, Eid MA, Creager MA, Goodney PP (2020). Epidemiology and risk of amputation in patients with diabetes mellitus and peripheral artery disease. Arteriosclerosis, thrombosis, and vascular biology.

[60] Goodney PP, Likosky DS, Cronenwett JL, England VSGoNN (2009). Predicting ambulation status one year after lower extremity bypass. Journal of vascular surgery.

[61] Waters RL, Perry J, Antonelli D, Hislop H (1976). Energy cost of walking of amputees: the influence of level of amputation. JBJS.

[62] Huang C, Jackson J, Moore N (1979). Amputation: Energy cost of ambulation. Archives of physical medicine and rehabilitation.

[63] Omololu A, Ogunlade S, Gopaldasani V (2008). The practice of traditional bone setting: training algorithm. Clinical orthopaedics and related research.

[64] Omololu B, Ogunlade S, Alonge T (2002). The complications seen from the treatment by traditional bonesetters. West African journal of medicine.

[65] Ekere AU, Echem RC (2011). Complications of fracture and dislocation treatment by traditional bone setters: A private practice experience. Nigerian Health Journal.

[66] Akinyoola A, Oginni L, Adegbehingbe O, Orimolade E, Ogundele O (2006). Causes of limb amputations in Nigerian children. West African Journal of Medicine.

[67] Yakubu A, Muhammad I, Mabogunje O (1995). Limb amputation in children in Zaria, Nigeria. Annals of tropical paediatrics.

